# Next-generation sequencing of the mitochondrial genome of *Macculochella peeli* (Percichthyoidei: Percichthyidae)

**DOI:** 10.1080/23802359.2020.1768953

**Published:** 2020-05-28

**Authors:** Chengfei Sun, Linhui Cheng, Kun He, Shilin Zhang, Fugui Li

**Affiliations:** a Key Laboratory of Tropical and Subtropical Fishery Resource Application and Cultivation, Ministry of Agriculture and Rural Affairs, Pearl River Fisheries Research Institute, Chinese Academy of Fishery Sciences, Guangzhou, China; b College of Animal Science and Techonology, Jiangxi Agricultural University, Nanchang, China

**Keywords:** *Maccullochella peeli*, mitogenome, phylogenetic tree

## Abstract

The *Maccullochella peeli* belongs to family Maccullochella, and is distributed in Australia and South America (mainly Argentina and Chile). In this paper, the complete mitochondrial genome of *M. peeli* was determined using next-generation sequencing. The whole mitogenome is a typical circular DNA molecule of 16,442 bp and contains 13 protein-coding genes, 22 transfer RNA genes, 2 ribosomal RNA genes, and a D-loop region, with the base composition of A 31.6%, G 14.3%, T 26.3%, and C 27.8%. Phylogenetic analysis showed that *M. peeli* was the nearest sister to *Macquaria australasica*. Our whole mitogenome presented here would be useful for further study of *M. peeli*.

The *Maccullochella peeli* belongs to the family Maccullochella and the species is distributed in Australia and South America (mainly Argentina and Chile) (Rowland 1983, 1989). Limited mitochondrial genome sequences have been reported from family Maccullochella. Here, we sequenced the complete mitochondrial genome of the adult fish, collected from Foshan in Guangdong, (latitude: 22°53′12.06′′N, longitude: 112°58′37.33′′E), Foshan Xinrong Fisheries Company, to provide reference information for further study on this species.

The typical specimen was deposited in the Jiangxi Agricultural University aquatic museum (M.peeli-001). Total genomic DNA was extracted from muscle tissue using the standard phenol-chloroform protocol (Barnett and Larson 2012). Then, the paired-end DNA library with an insert size of 400 bp was constructed and sequenced by Illumina X-ten with 150 bp in read length (Zhang et al. 2018). The de novo assembly of the mitochondrial genome was assembled by NOVOplasty (Dierckxsens et al. 2016).

The complete mitochondrial genome of *M. peeli* (GenBank Accession no. MN842722, 16,442 bp) contains 13 protein-coding genes, 22 transfer RNA genes, 2 ribosomal RNA genes, and a D-loop region. The whole base composition of the mitogenome is showed as follows: A 31.6%, G 14.3%, T 26.3%, and C 27.8%. The total length of the 13 protein-coding genes is 11,435 bp, all of which are encoded on the heavy strand, except for *nad6*, which was in the light strand.

We performed a phylogenetic analysis of *M. peeli* species and *Lepomis gibbosus* outgroup species, *Siniperca chuatsi*, *Micropterus salmoides*, *Banjos banjos*, and *Macquaria australasica* based on 13 protein-coding genes sequences using maximum-likelihood method implemented in the RAxML (Silvestro and Michalak 2012). *Macquaria australasicawas* was the nearest sister to *M. peeli* (Figure 1). The complete mitochondrial genome of *M. peeli* we determined would be useful in systematics and population genetics.

**Figure 1.**
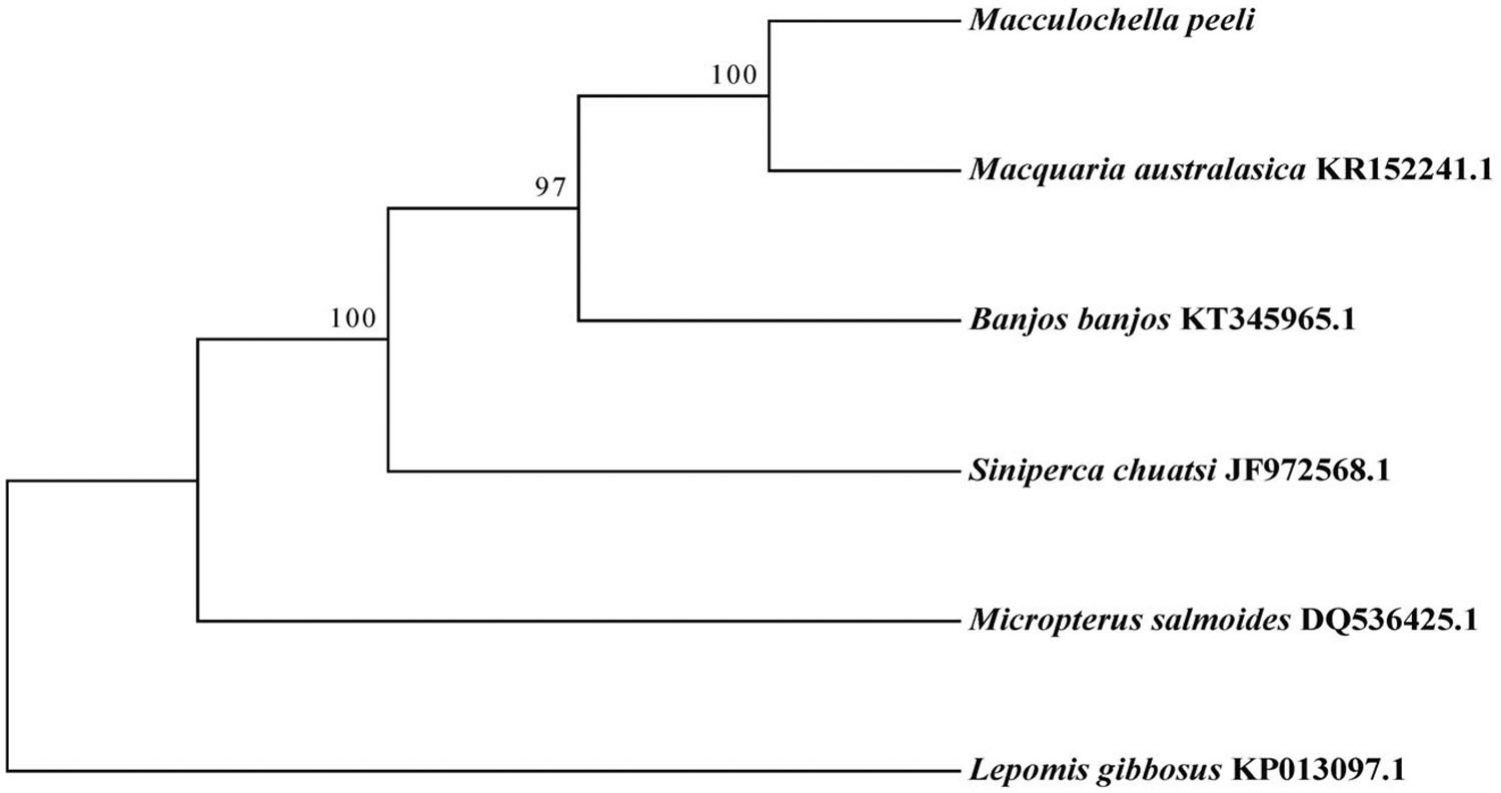
Phylogenetic tree generated using the maximum-likelihood method based on 13 protein-coding genes.

## Data Availability

The data that support the findings of this study are openly available in NCBI at https://www.ncbi.nlm.nih.gov/nuccore/MN842722, reference number MN842722.
